# Neurocognitive Mechanism of Perceptual Errors in Radiologists: Results of Pilot Studies

**DOI:** 10.1177/26331055261470384

**Published:** 2026-07-21

**Authors:** Michael A. Bruno, Elizabeth A. Krupinski, Scott C. Bunce, Grayson L. Baird, Caitlin Mills, Prasanna R. Karunanayaka, Howard Egeth, Ryan Chang, Ross Cottrill, Samuel Jump, Krishnankutty Sathian, Timothy J. Mosher

**Affiliations:** 1 Department of Radiology, Milton S. Hershey Medical Center, 12311 Penn State Health, Hershey, PA, USA; 2 Neuroscience Institute, 12310 Penn State College of Medicine, Hershey, PA, USA; 3 Department of Radiology, 12239 Emory University School of Medicine, Atlanta, GA, USA; 4 Department of Radiology, Rhode Island Hospital, 6752 Brown University, Providence, RI, USA; 5 Department of Educational Psychology, 5635 University of Minnesota Twin Cities, Minneapolis, MN, USA; 6 Department of Psychological & Brain Sciences, 1466 Johns Hopkins University, Baltimore, MD, USA; 7 Mallincrodt Institute of Radiology, Washington University, St. Louis, MO, USA; 8 Dept. of Pediatrics, Duke University Medical Center, Durham, NC, USA

**Keywords:** functional connectivity, network neuroscience, radiologist performance, perceptual error, default mode network

## Abstract

**Background:**

The most prevalent type of radiologist error is failing to detect abnormalities on images, the so-called “perceptual error.” The prevalence of this type of false-negative (FN) error remains essentially unchanged since it was first described in 1949. Reducing this error rate would be highly beneficial for patients who often depend on radiological observations for a timely diagnosis.

**Purpose:**

To identify a potential neurocognitive mechanism contributing to radiologists’ perceptual errors, in order to inform intervention strategies to reduce such errors in practice. These experiments evaluated the relationship between brain network activation states and radiologists’ perceptual errors on two distinct visual tasks utilizing functional MRI (fMRI) and functional Near Infrared Spectroscopy (fNIRS).

**Materials and Methods:**

A prospective, pilot study was carried out on a small number of radiologist participants utilizing two distinct types of visual tasks while monitoring activity in two targeted brain networks, the default mode network (DMN) and the “executive” frontoparietal network (FPN), to test the hypothesis that simultaneous co-activation of these networks produces an error-prone neurocognitive state (EPS). A third experiment using fNIRS alone was an observational study of subjects’ neurocognitive states during their usual practice to determine the baseline prevalence of the EPS in the clinical setting.

**Results:**

An approximately threefold increased risk of FN perceptual errors (misses) on the visual tasks was observed in the presence of simultaneous co-activation of elements of the Default Mode Network (DMN) and Frontoparietal Network (FPN), which supports the “error-prone state” (EPS) hypothesis. EPS prevalence declined with participant age.

**Conclusion:**

Our results suggest that dynamic interactions between the Default Mode Network (DMN) and Frontoparietal Network (FPN) create a unique error-prone state (EPS), associated with an approximately threefold increased risk of perceptual error in radiologists. These results suggest potential intervention strategies for perceptual error, the largest class of radiologist errors in practice.

## Key Results

1. Transitory episodes of a discrete neurocognitive state involving co-activation of the Default Mode Network and Frontoparietal Network were observed to occur spontaneously in radiologists as they performed specific visual tasks and in actual clinical settings.
2. There was an approximately threefold increased risk of a participant experiencing a perceptual error during an episode of this state, as most of the FN errors that were observed occurred during these brief episodes (p < 0.01).
3. In an observational experiment of radiologists performing their usual tasks in their actual clinical setting, there was a highly significant anti-correlation of the prevalence of the error-prone neurocognitive state (EPS) with subject age (p < 0.001).

## Introduction

Radiologists frequently fail to visually perceive abnormalities that are later shown to have been present on images, including many that are readily detected in retrospect. This type of error, known as “perceptual error,” is by far the most common error type for radiologists, accounting for the majority of radiologists’ errors in practice.^1,2^ In contrast, relatively few diagnostic errors are due to radiologists’ misinterpretation of detected findings, cognitive biases or clinical knowledge deficits.^3,4^ Since first described by Dr. Leo Henry Garland in the late 1940’s,^5^ the prevalence of perceptual error in radiology has not appreciably changed.^6^ Dr Garland’s pioneering work in the late 1940s and early 1950s showed that a low (but nonzero) rate of errors appeared to be an intractable reality in radiological practice. He recommended double-reading as a potential solution. Subsequent research on errors confirmed and extended Garland’s findings. While radiologist error rates as low as 3-4% have been reported in the setting of low prevalence of positive findings, higher error rates, on the order of 30% are typical when radiologists are presented with test cases that have a higher prevalence of abnormalities, with some studies showing up to 70% of lung cancers missed on chest x-rays and 39% on CT scans.^7^ The etiology of these common errors of omission is likely the result of both visuo-perceptual and cognitive factors acting together in complex ways.^8^ Such errors appear to be due to a basic human factor, as they have not been mitigated by substantial improvements in imaging technology and radiological knowledge over the past several decades.

**Figure 1.**
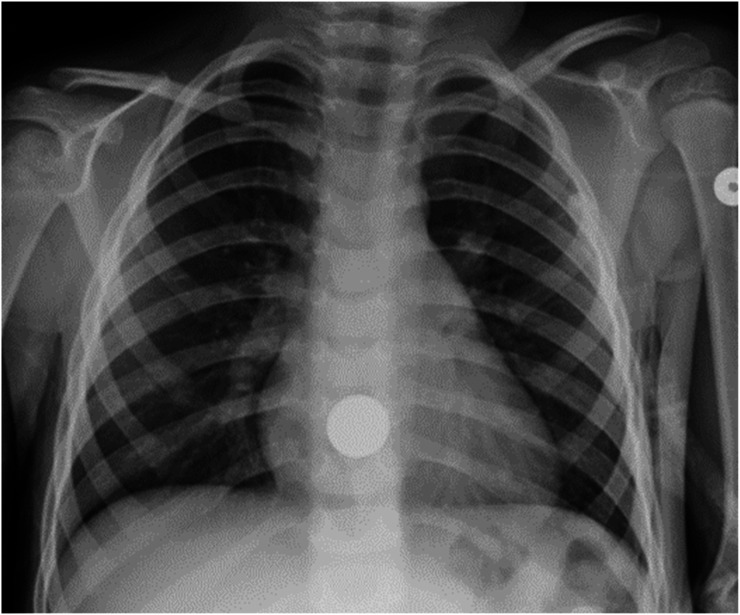
A chest radiograph of a child obtained to rule out a swallowed coin. The presence of the coin within the esophagus is readily apparent on the image, but was missed twice by a highly experienced and skilled pediatric radiologist. This case illustrates that even very conspicuous abnormalities can be overlooked by highly skilled observers (from Reference 2, used with permission)

It is an attractive hypothesis that many radiologists’ perceptual errors could be the result of natural fluctuations in attention or task engagement related to human operator neurocognitive states that lead to momentary lapses in attention or receptiveness to visual stimuli. Critically, such inattentive states can occur without operator awareness,^9^ making them difficult or impossible to detect in real-time, although they are often readily apparent in retrospect (see Figure 1). When such errors involve highly conspicuous abnormalities, radiologists are often at a loss to explain how such a finding could have been missed. When errors of this type lead to protracted delays in diagnosis, they can have devastating consequences for patients. For example, in patients with many types of cancer, delays in diagnosis can greatly restrict options for treatment and lead to worsened outcomes.

**Figure 2.**
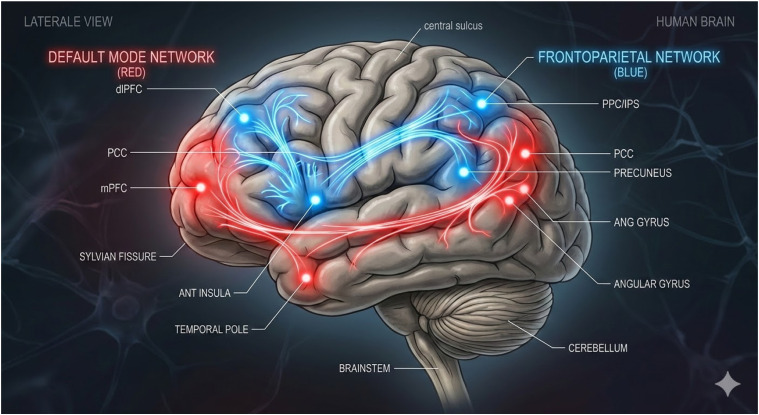
A graphic representation of the anatomic locations of the nuclei and connective pathways of the default mode network (shown in red) and the Frontoparietal Network (shown in blue). Under normal circumstances, in the resting state, only one or the other of these two networks will be active

An emerging literature within the broader field of network neuroscience has described a particular neurocognitive state, which we have called an “Error-Prone State (EPS),” that results from interactions between two well-described networks of cortical brain regions.^10,11^ The first is the “Default Mode Network,” or DMN, which was first identified by Raichle *et al,*^12,13^ and which is believed to underly internally-focused modes of thought, and the other is the Fronto-parietal network (FPN), which is believed to underly externally-directed thought and tasks requiring attention to outside stimuli.^14^ The anatomic location of the major components of each of these networks is illustrated in Figure 2.

It has been observed that the DMN generally acts in opposition to the FPN, with the two networks alternating in periods of activation and suppression during the course of a normal day. Extensive observational research using BOLD *f*MRI has shown that, under usual circumstances, activity within either one of these two networks is anti-correlated with activity in the other. Indeed, human subjects are observed to “toggle” between them as they begin and finish externally-directed tasks. The “toggle” function is apparently under control of additional cortical networks.^15^ Researchers have, however, identified a particular episodic or transient neurocognitive state wherein there is simultaneous co-activation of these two functional brain networks, creating a hybrid DMN+FPN neurocognitive state. It has further been shown that this state is associated with an apparent decoupling of sensory inputs from cognitive awareness.^16^ This state can occupy up to 30% of a person’s waking hours and is generally not under volitional control.^10,17-19^ Subjects are notably less receptive to visual stimuli at these times, despite the appearance of continuing to attend to and search the input.^11,20,21^ It has been suggested that when the two networks are simultaneously active the DMN effectively “hijacks” the resources of the FPN, including higher-order cognitive processing and working memory, and also effectively suppresses activity in cortical areas that process sensory stimuli, such as the primary visual and auditory cortices, perhaps to protect a train of internal thought from being interrupted by external distractions from auditory or visual stimuli.^22^ The perceptual decoupling hypothesis also provides a plausible mechanistic explanation for earlier findings from research performed in the 1970s using eye-tracking, which showed that radiologists looked directly at 70% of unreported lung nodules in chest radiographs, but subsequently had no awareness or recollection of having seen them.^23^ The exact neurophysiological mechanisms of that subtend this interaction remain unknown.

The significance of this intermittently occurring and transient brain phenomenon from an evolutionary standpoint is unknown; it is likely a feature of a higher-level human mental function which is not, as yet, understood. It does, however, appear to increase the risk of non-detection (false-negative) errors by Radiologists. Unlike conditions of fatigue, when in this altered mental state radiologists appear to be searching effectively, both to outside observers and from their own subjective experience, however conspicuous abnormalities/visual targets on images are neither detected nor incorporated into working memory. Such false-negative errors can result in substantial delays in diagnosis for patients, which can sometimes have devastating consequences for the patient’s health and treatment.

This pilot study involving a small cohort of expert participants aimed to test the hypothesis that transient episodes of this well-described hybrid neurocognitive state increase the risk of perceptual error in radiologists performing visual tasks, possibly due to its associated phenomenon of perceptual decoupling. In this work we refer to this neurocognitive phenomenon as the “Error-Prone State,” or EPS, and suggest that it might provide a mechanistic explanation for many (perhaps a majority) of radiologists’ perceptual errors in practice.

To test this hypothesis, we have carried out three experiments that evaluated the occurrence of the EPS in experienced radiologists performing two very distinct visual tasks, one involving continuous participant attention and another involving visual search. We also observed the EPS in the radiologists’ actual work setting. Our goal was to explore whether EPS is a significant risk factor for perceptual error in our subjects, to determine the prevalence of EPS under each of these conditions, and to evaluate the feasibility of detecting EPS using a relatively unobtrusive measure as a path for future work aimed at interventions to reduce perceptual errors.

The results of our study affirmed that radiologists experience seemingly randomly-occurring episodes of a discrete, transient neurocognitive state, which we have termed the “Error-Prone State,” (EPS), and which may provide a potential mechanism for radiologists’ perceptual errors in practice. While in the EPS, our radiologist participants were much more susceptible to false-negative errors, i.e., those where they fail to perceive the targets that they were looking for on the images they viewed. In this pilot study we have also shown that the EPS can be detected reliably and unobtrusively in the normal clinical setting using fNIRS, a noninvasive and inobtrusive measure. This, in turn, suggests that detection of the EPS in radiologists as they work could possibly serve as a useful biomarker for point-of-care strategies aimed at reducing this class of errors in practice. Since “look-but-fail-to-see” perceptual errors are the most common error-type for radiologists, accounting for 70-80% of their total errors,^2^ the impact of such an intervention could potentially be substantial, if results can be validated in larger studies and if engineering and other challenges to clinical translation of this work can be overcome in future work. As the utilization of medical imaging has increased dramatically in recent years, radiologists currently play a key role in the diagnosis of many patients, including nearly all patients admitted to emergency departments and hospitals. Improving radiologist performance would therefore translate to many fewer diagnostic errors, which have been suggested to be a leading cause of death in the United States.

## Ethical Considerations

Our study was approved by the Penn State College of Medicine Institutional Review Board for protection of human subjects, approval No. 00007773, on August 16, 2018, and has been re-reviewed and reapproved annually since that time. The most recent re-approval was granted on April 10, 2026. Full written informed consent was obtained from each subject for participation in the study and for data publication before inclusion in the study, and all were free to withdraw from the study at any time, and told they had the option to review their own data if desired. The authors retained full control of the data and determined all of the content which has been submitted for publication.

## Materials and Methods

Three experiments were conducted in our pilot study, utilizing functional MRI (fMRI) and functional near-infrared spectroscopic imaging (f*NIRS*) to assess brain states of our radiologist subjects as they carried out various cognitive tasks. For the experiments involving fNIRS we received a free loan of fNIRS equipment along with customized analytical software from the NIRx Corporation (Berlin, Germany). Subjects were all fully-trained radiologists currently in active practice, all of whom work in a single center. A range of ages was sought; however the lower-age boundary is constrained by the number of years required for the training of radiologists, such that most radiologists begin their careers at the age of 33 years or older. Retired radiologists were not included. The study was conducted in June through August of 2023 at Penn State Hershey, in Hershey, PA, USA. The participant group was consecutively enrolled from June 13, 2023 through August 11, 2023.

Although fMRI is the recognized gold standard for study of brain networks, and was the modality from which the Default Mode Network was originally discovered in the resting state,^12^ fNIRS offers a number of advantages in that it is portable, relatively unobtrusive, and highly sensitive and specific for regional brain activation states. In our pilot study, fNIRS was performed using a NIRx Scout™ system (NIRx Medical Technologies, LLC, Berlin, Germany), using a fully MRI-compatible array of optodes. fNIRS is a well-established noninvasive and portable means to perform functional brain imaging using the BOLD effect, based on the absorption of light in the near-infrared spectrum, from 700 to 1300 nm wavelength. Biological tissues (scalp, skull, meninges) are relatively transparent at these wavelengths, and so the absorption spectra are largely dependent on the oxygenation and deoxygenation states of hemoglobin and the redox state of cytochrome c oxidase in mitochondria. fNIRS can measure concentration changes in oxygenated (oxy-Hb) and deoxygenated (deoxy-Hb) hemoglobin within the human brain with high accuracy over short time-frames, and the technique is commonly used in neuroimaging studies and also in limited clinical applications. The basic principle is similar to fMRI, however the spatial resolution is significantly lower, and signal can only be derived from more superficial brain areas within the optical path, generally at depths less than 25mm.^24^ For both experiments 1 and 3, an optode array optimized for the prefrontal cortex was utilized, with 8 laser light sources and 11 detectors, held to the scalp in standard position by a specialized cap designed for this purpose.

**Figure 3.**
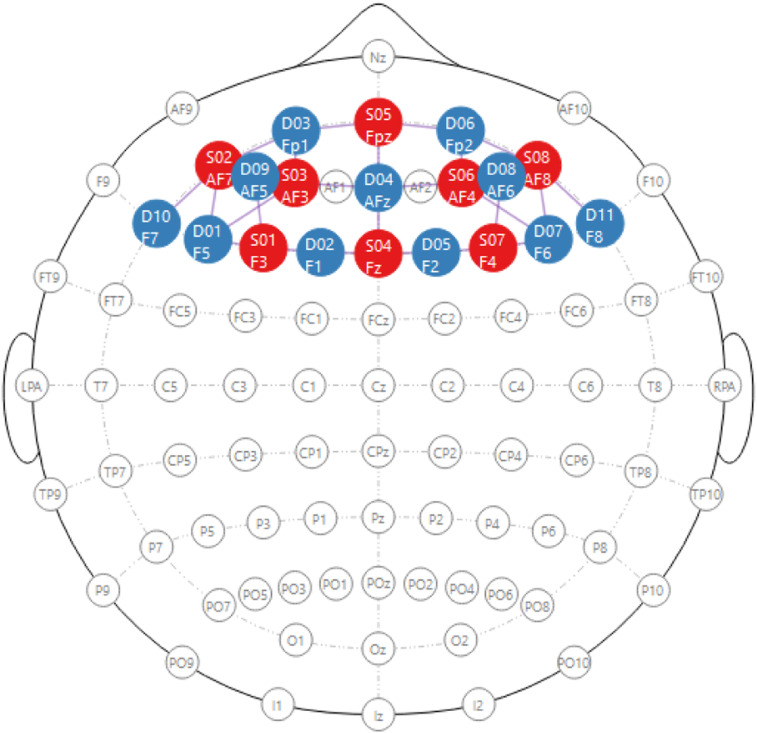
The ‘Optode Montage” showing the physical arrangement of laser light sources (red dots) and detectors (blue dots) used in our *fNIRS* experiments

Figure 3 illustrates the arrangement of these elements, which is often referred to as the optode “montage.” Data were acquired using NIRStar™ software and analyzed using NIRSlab™ a Matlab-based application developed for this purpose SUNY Downstate University, Brooklyn, NY, and licensed by NIRx (which was provided for our pilot study free of charge).

In the first experiment, 10 radiologists performed a visual task requiring their continuous attention for 15 minutes, divided into two 450-second sessions. Subjects included 8 males and 2 females, ranging in age from 37-65 (mean = 49.5). All but one were right-handed. The subjects were instructed to watch a small, gray disc as it slowly meandered across their viewing screen on a textured grayscale background reminiscent of the appearance of x-ray images. At pseudo-random times, and without warning, the luminosity of the disc abruptly changed, at which time they were to press a button indicating that they had detected an event. By design, this continuous attention task (CAT) uses subtle luminosity changes. These visual events are readily detectable, but only if the subject is continuously tracking the disk and paying close attention to it. The task is designed such that it is virtually impossible for a subject to determine whether a change-event occurred retrospectively. The single visual target is always in plain view and moves across the screen very slowly, such that the task does not involve visual search. When the subject detects a change, they signaled by pushing a button, allowing measurement of response times. By measuring accuracy, *e.g.,* successful detection and response times, it was possible to detect lapses in the subjects’ attention with high temporal precision, and correct responses were detected in as little as 550 ms. As a cut-off value, any response recorded within 3 seconds of an event was accepted; after that time, any further-delayed response was counted as a miss. Responses that were not temporally related to an event were counted as FP. The task was not in itself difficult or challenging but required the continuous attention of the subject in order to detect the visual events.

For assessment of subjects’ neurocognitive states during the task, we utilized simultaneous whole-brain *f*MRI and *f*NIRS of the prefrontal cortex (PFC), using an MRI-compatible *f*NIRS system (NIRx Scout, NirX Medical Technologies, LLC, Berlin). *f*MRI was performed using a 3.0T Siemens PrismaFit MRI system (Siemens, Erlangen, Germany). For the *f*MRI analysis, the time course of activation BOLD (OxyHb) signal was correlated for the DMN and FPN to compare temporal behaviors to detect co-activation events, defined as a positive (in-phase) dynamic correlation between the two normally out-of-phase (anticorrelated) networks using scale-independent amplitudes of activity. Independent analysis was performed for the 8 of these subjects for which *f*NIRS data were also available, and *f*NIRS and *f*MRI results were compared for these 8 subjects in order to validate the use of *f*NIRS, which was limited to the Prefrontal Cortex (PFC), against the whole-brain *f*MRI “gold standard.”

Functional MRI (*f*MRI) was performed on a 3.0T Siemens PrismaFit system with a 64-channel head coil and two-channel transmit capability (Erlangen, Germany). Computational support includes a 32- processor cluster. An Eloquence™ System for functional MR imaging (Invivo Corp, FL, USA) including an LCD visual display, audio system, button-response unit and e-prime software for paradigm creation and visual display, subject management, precise delivery of stimuli and behavioral data analysis (Brain Voyager Package). The system is also equipped with an EyeLink 1000 Plus System (SR Research, Ottawa, ON, Canada). The SR Eyelink Host PC performs real-time eye-tracking at 250, 500, 100, or 2000 samples per second while computing true gaze position on the display viewed by the participant. The system also performs analysis of eye-motion events, such as saccades, blinks and fixations. fMRI acquisition parameters utilized a TR of 8.6 ms, TE of 4.00 ms, with a 7mm slice thickness, with 2 averages and a flip angle of 20 degrees, with a phase resolution of 91% and a bandwidth of 320 Hz/Px. Resting-state acquisition was done for several seconds prior to onset of the visual paradigm.

Each 450-second run had 50 visual events to be detected, with a pseudorandom variable inter-stimulus interval. The timing of FN “miss” errors was correlated to both the *f*MRI and *f*NIRS to ascertain to what extent subject errors correlated with the “target” EPS, and to assess the level of concordance between the two techniques, with the goal to validate the later use of only *f*NIRS in the clinical setting. The rationale for using *f*NIRS, which is limited to visualizing BOLD signal only the relatively superficial PFC components of the DMN and FPN, was based on the well-established observation that the several brain areas that comprise each network demonstrate tightly-coupled, coordinated patterns of activation, such that it would be unusual for some components of either the DMN or FPN to be active when others are not; this is a fundamental feature defining these networks.^12,13^ We therefore predicted that it would be most likely that activation of the PFC components of the DMN and FPN alone would be highly predictive of the behavior of the entire network. The degree of correlation was measurable by comparison of the simultaneously-acquired and independently analyzed *f*NIRS and *f*MRI data.

In Experiment 1, as noted above, we performed fMRI and fNIRS *simultaneously* as subjects performed the continuous-attention (CAT) task. A signal from the e-prime system launched the visual task paradigm and synchronized data acquisition for both the fMRI and fNIRS systems, functioning independently. The CAT task itself required the participants to engage for 15 minutes, divided into two 450-second sessions, requiring subjects’ continuous attention for 7.5 minutes at a time. All subjects’ sessions were scheduled to begin between 11:00 a.m. and 4:00 p.m., to minimize the potentially confounding effects of diurnal or workday fatigue.

The fMRI data were analyzed independently of the fNIRS data, using dedicated analytical software developed by one of the investigators (PRK) for this purpose. It quantifies regional brain activity on a relative scale for the pre-identified cortical brain regions which comprise the two targeted networks, the DMN and FPN, in a manner similar to prior publications.^25^ Brain regions measured for the DMN components in the fMRI analysis included the ventromedial and dorsomedial prefrontal cortex, the posterior cingulate cortex, the posterior inferior parietal lobule, and the lateral temporal cortex. FPN brain regions included the anterior prefrontal cortex, dorsolateral prefrontal cortex, anterior cingulate, anterior inferior parietal lobule, and anterior insular cortex (see Figure 2). Each subject was individually analyzed at the network level, comparing the relative amplitudes of activation of the DMN and FPN networks. Although any degree of positive correlation of the two networks would support our hypothesis, for added rigor, a cutoff value for the Pearson coefficient of correlation between the activity amplitudes of the two networks was set at 0.4. In development of our protocol a range of thresholds were evaluated for this purpose (i.e., from 0.3 – 0.6) and all yielded similar results. The choice of >0.4 represented a compromise that served to optimize the balance of sensitivity with specificity when determining an EPS was present. Our analysis excluded networks other than the DMN and FPN.

For fNIRS, BOLD assessment was limited to the prefrontal cortex. This method has less spatial resolution than *f*MRI. We were, however, able to discriminate cortical activation (OxyHb signal) in the medial PFC, which is a component of the DMN, from the dorsolateral PFC, which is a component of the FPN. When high-amplitude OxyHb signal on the order of 10-20 mM of oxyHb was seen in both PFC cortical regions, it was scored as an EPS event. BOLD signal was measured within these distinct PFC areas during the antecedent 10 sec prior to each visual task event, which is within the temporal resolution of the *f*NIRS method. In this way, we were able to [1] determine when attentional lapses had occurred [2] determine the underlying brain states at those times to determine whether the EPS (co-activation of both DMN + FPN) was present, on either fMRI and/or fNIRS, and [3] compare the two.

As discussed above, we expected that the fNIRS sampling of only one of several components of each network (medial PFC for the DMN and dorsolateral PFC for the FPN) would be likely to correlate well with the whole-brain multinuclear analysis done by fMRI. One aim of Experiment 1 was to evaluate the level of concordance between the two modalities, given that *f*NIRS utilizes a comparatively limited brain sample. We also determined the fraction of time subjects spent in the EPS during this task, for both the fMRI and fNIRS. As an added quality control, in order to verify that the presence of the fNIRS system did not degrade the fMRI signal, the CAT task was run once with both fNIRS and fMRI and then again using fMRI only, without the fNIRS apparatus in place. Signal quality was compared between the two runs to assess whether the fNIRS detector system caused any unforeseen artifacts or BOLD signal loss on fMRI. fNIRS data quality metrics (noise and physiological measures) were observed to be excellent for both runs.

The second experiment utilized fMRI alone (no fNIRS) with the addition of concurrent eye-tracking, using a conventional psychophysical visual search task. This experiment was designed to determine whether the association of EPS and error differed when the subjects were actively searching the images for a target, in order to generalize the results beyond sustained attention. Visual search is fundamental to the practice of radiology. Eye-tracking was performed using an SR Eyelink™ system (SR Research, Ottaway, ON, Canada) in order to determine whether the subjects looked at the targets with their central, foveal vision as part of their decision-making process.

**Figure 4.**
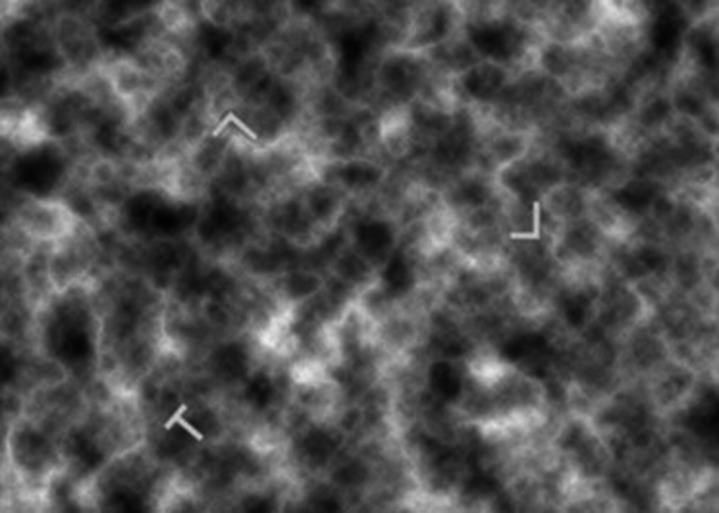
Sample T/L Image. A target ‘T’ is seen in the lower left. In the experiment, a an observational frame may or may not have included a “T” target

For this experiment a total of 6 Radiologist subjects (five male, one female, all right-handed) were tasked with detection of a target grayscale “T” on a textured grayscale background among numerous “L” distractors (Figure 4). In preparatory work using multiple radiologist and non-radiologist volunteers, the task was designed to be moderately difficult (on the basis of both objective subject performance and subjective measures). Task difficulty was adjusted by manipulation of the opacity/translucency of the T’s and L’s, altering the number of “L” distractors, as well as limiting the time subjects were allowed to view each task to 10 seconds. Subjects were engaged for approximately 15 minutes in two 450-second sessions. Each trial contained multiple “L” distractors and may or may not have included a “T” target. Each 450-second session included 60 T/L observational frames. If a “T” was detected, the subject would indicate this by pressing a button, and they could also indicate when they believed that no “T” was present, by pressing a different button.

Detection accuracy (FP and misses) was assessed, along with reaction times, and eye-tracking allowed us to determine whether the subject visually fixated on a target. Subjects performed the task within the MRI bore, and their neurocognitive states were assessed by *f*MRI.

As before, fMRI acquisition parameters utilized a TR of 8.6 ms, TE of 4.00 ms, with a 7mm slice thickness, with 2 averages and a flip angle of 20 degrees, with a phase resolution of 91% and a bandwidth of 320 Hz/Px. Resting-state acquisition was done for several seconds prior to onset of the visual paradigm. Each subject performed the task twice, in 450-second sessions. Experiment 2 allowed us to evaluate the EPS/Perceptual error hypothesis for the situation of an active visual search by the participant, which differs from the task of continuous attention in Experiment 1. We also determined the fraction of time subjects spent in the EPS during this task.

A third experiment used fNIRS alone (NIRx Scout, NirX Medical Technologies, LLC, Berlin) and took place in the actual clinical setting. This was an observational study using 9 radiologists (4 men and 5 women) to verify the feasibility of using portable fNIRS technology to assess radiologists’ neurocognitive states as they performed their usual interpretive work tasks. We determined what fraction of the radiologists’ time was in the high-risk EPS target-state as they worked, using the same fNIRS criteria as in the CAT task experiment. The prevalence of EPS was correlated with subject age. As before, the presence of the EPS was inferred when there was simultaneous activation of the ventromedial PFC (a component of the DMN) and the dorsolateral PFC (a component of the FPN). Our expectation was that, since the EPS is task-independent, we should observe an overall prevalence of the EPS state in the clinical setting that is similar to fMRI and fNIRS our other two experiments.

There was some overlap of participants between these three experiments, with three subjects having participated in at least 2 of the experiments and one subject having participated in all three.

## Statistical Analysis

All statistical modeling for our data analysis was accomplished using SAS Software 9.4 with the GLIMMIX procedure. For our first two experiments, generalized linear modeling (GLM) was used to model state frequency and duration assuming a Poisson and binomial distribution, respectively. False negative rate by state was examined using generalized linear mixed modeling assuming a binary distribution, where observations were nested within radiologists (radiologist were a random effect). Error events were examined as a function of the absolute ratio between DMN and FPN as a natural cubic spline using generalized linear mixed modeling (GLMM) assuming a binary distribution with a logit link function, where each participant had their own slope and intercept. As spline modeling is non-linear, no parameters could be estimated. In the analysis of the results of Experiment 3, EPS was modeled by age using GLM assuming a binomial distribution.

## Results

### (1) Experiment 1: Continuous Attention Task

In the first experiment using the continuous attention task (CAT), the *f*MRI data from one (male) subject was corrupted and could not be analyzed, leaving 9 fMRI subjects for whom excellent quality fMRI and task-level data were available for analysis. Of these, there was one (female) subject for which a malfunction degraded the* f*NIRS data, so that technically excellent quality fNIRS data were ultimately obtained for 8 subjects. The average error (miss) rate per subject was 10% (range 2% - 26%). Correct response times ranged from 550ms - 2.0 seconds (mean = 1.2 sec). There were a total of 52 FN and 15 FP errors in our 9 subjects.

**Figure 5.**
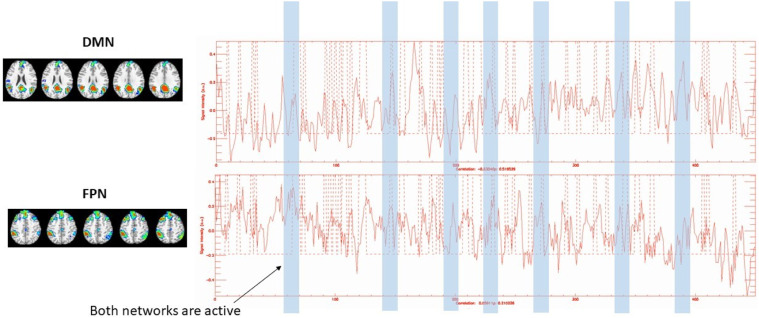
Representative *f*MRI data from one subject radiologist showing the relative amplitude time course of network activation as a function of time during the 450-second CAT task for DMN (upper) and FPN (lower). Y-axis = relative signal intensity amplitude of network activation by *f*MRI (BOLD) and X-axis = time in seconds (0-450). These two networks were observed to be generally opposed, as expected, however several brief episodes of co-activation of both networks were observed (blue shaded regions). These episodes correspond to the EPS. For this subject, 7 such episodes were detected during the session

Irregular, aperiodic brief episodes of EPS were observed, as illustrated with blue shading in Figure 5, below, which is of a single-subject from Experiment 1. During the CAT visual task, on both fMRI and fNIRS, cortical activation patterns were observed to shift between periods of activity in the FPN, alternating with periods of DMN activation (generally opposed), punctuated by brief, episodes where subjects were observed to be in the target EPS state*, i.e.,* where both networks were simultaneously active. The number and duration of spontaneous episodes of EPS detected varied, ranging from 6 – 12 in each 15-minute block, with an average of 8.5 spontaneous EPS events, most lasting on the order of 2-4 seconds. Their temporal occurrence appeared to be spontaneous, aperiodic and irregular, with no apparent relationship to the visual task.

Subjects were found to be in the EPS approximately 24 – 35% of the session time. As predicted, the EPS state was found to correlate strongly with FN errors but not FP errors. Although FN errors were relatively few, and episodes of EPS were relatively brief, a total of 37 FN errors (comprising 71% of the total 52 FN errors) were found to have occurred during EPS episodes by fMRI (all 9 subjects) and 29 out of 39 FN errors occurred during EPS by *f*NIRS criteria in the 8 subjects for which fNIRS data were collected. This was a highly significant finding, p<0.01 (generalized linear modeling). FN errors were also seen at times of state transition or during periods of DMN activation without FPN activation. These results are tabulated below.

**Figure 6.**
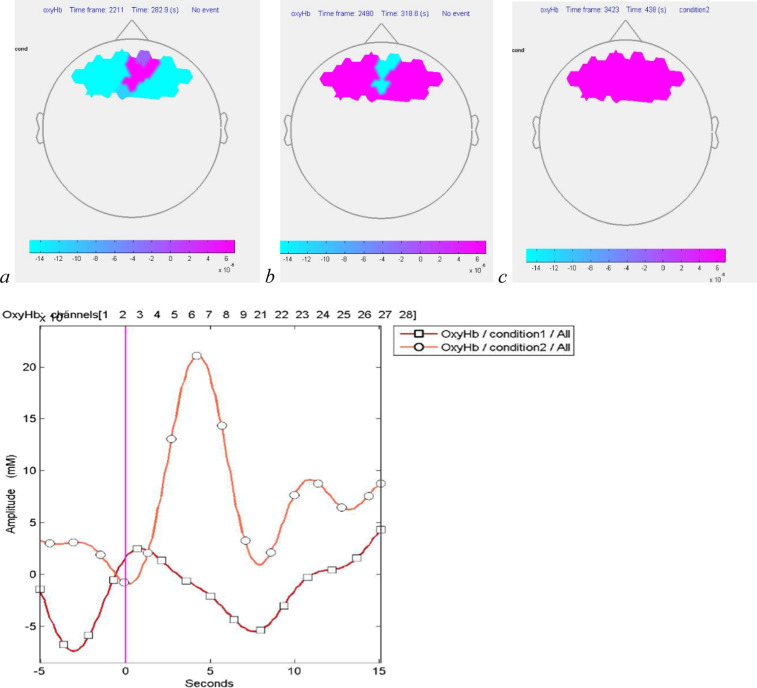
Representative spatial activation maps from *f*NIRS of BOLD (OxyHb) signal in a single subject, obtained while performing the CAT task while simultaneously undergoing *f*NIRS and *f*MRI. Maps depict signal within the channels corresponding to the medial (A) and dorsolateral (B) PFC activation occurring between tasks, corresponding to fluctuations between DMN and FPN activity as shown on *f*MRI, and (C) in EPS, with both brain regions active. FN errors (condition 2) were highly correlated with EPS for both *f*MRI and *f*NIRS (p < 0.01). Figure 6(D) is a signal-amplitude vs. time plot from experiment 1. Block-average plot of OxyHb levels from *f*NIRS in one subject corresponding to medial PFC channels of both cerebral hemispheres (DMN) during successful event detections (condition 1) and missed event detections (condition 2), corresponding to an episode of EPS. This plot illustrates the robust discriminatory ability of *fNIRS* to detect physiological activity within selected areas of PFC. Medial PFC areas (DMN) are highly activated during condition 2 (FN errors) compared to condition 1 (TP), as predicted. Dorsilateral PFC (a component of the FPN) was also active at this time. Perceptual errors did not correlate with DMN activation alone

Representative fNIRS results from Experiment 1 are shown as Figure 6, below. fNIRS was able to spatially discriminate activity between the two adjacent PFC areas (medial vs. lateral PFC) and identify their distinct activation states (Figures 6A,B) and detect EPS (Figure 6C) with a physiologically robust BOLD signal response (Figure 6D). There was 73% overall concordance between the fMRI results of network activation dynamics and the corresponding fNIRS results, including frequency, timing and duration of EPS episodes, and there was agreement between both modalities in 67% of the detected FN+EPS errors.

### (2) Experiment 2: Visual Search Task, With Eye-Tracking

**Figure 7.**
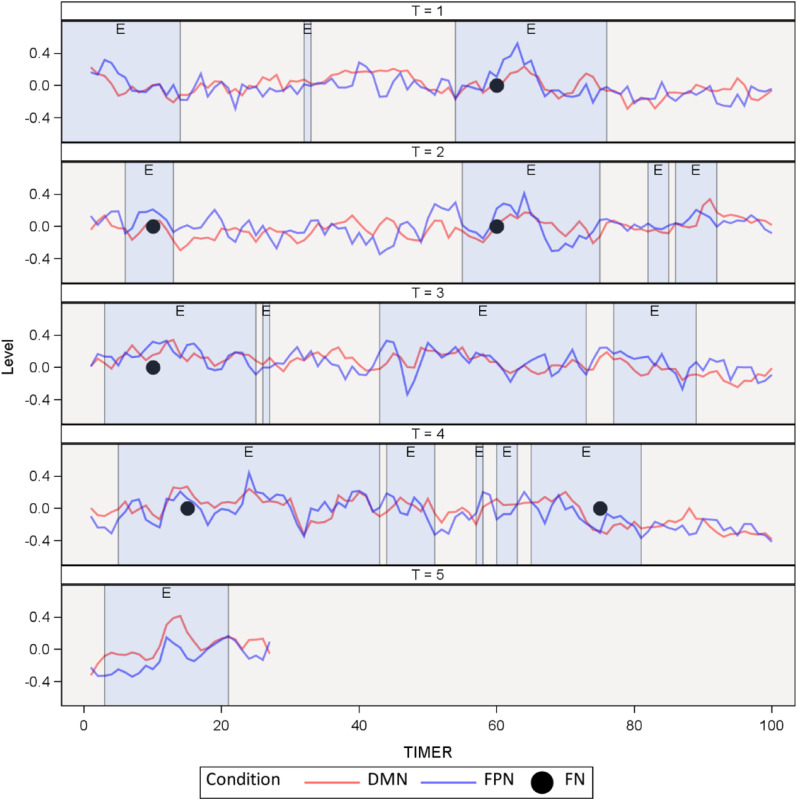
Representative fMRI data from one radiologist during the T/L search task, showing a 330 second sample within the time course of network activation (*p*<0.01) during a trial session. Curves reflect the levels of activation within the DMN (red) and FPN (blue). When the Pearson Coefficient of correlation between the two networks exceeds the target threshold of 0.4, an EPS event (E, blue-shading) is indicated. As before, activation in each of the two networks was generally anti-correlated, but with several brief and variable episodes of simultaneous co-activation (blue shaded regions) occurring. For this subject, 17 such episodes are shown, of varying durations. Black dots indicate the timing of FN error events, when the subject failed to detect the “T” target. Two of the FN errors were noted to have occurred despite the subject visually fixating on the “T” target. This subject also had two FP errors (not shown) which were unrelated to episodes of EPS. Y-axis = relative amplitude of network activations for DMN and FPN. X-axis = time in seconds (0-400)

In the second experiment, 6 subjects performed a visual search task using eye-tracking with fMRI. There were a total of 28 FN and 2FP errors observed, from a total of 360 trials, for an overall error rate of 7.7% across all subjects. Individually, subjects made 4.3 FN errors per session on average (range 2-7). Only one subject experienced any FP errors. Of 28 FN errors, there were 13 where the subjects had visually fixated on the target as shown by eye-tracking. Within a 450-second window containing 60 trials, the average number of EPS episodes per subject was 11 (95% CI, range 9.5-12.7) and the average fraction of time spent in EPS was 28.9% (95% CI, range 24.1% - 34.24%), or approximately 4.3 minutes. In considering the relative risk of FN errors comparing the EPS to non-EPS neurocognitive states, the average FN rate with 60 trials was 10.75% (95% CI, range 5.06 - 21.42) when not in EPS and 30.33% (95% CI, range 19.82 - 43.40) when in EPS, an odds increase of 3.61 (95% CI, range 1.39 - 9.35), with *p*=.0087. This shows that EPS may be a substantial risk factor for FN errors for this task. There was no evidence of any difference between FN errors whether eye-tracking showed fixation on the “T” target. There were only two FP errors, however, which both occurred in the same subject. These were unrelated to EPS. Table 1 summarizes the correlation of FN events with EPS in Experiments #1 and 2. A typical result is illustrated in Figure 7, below.

**Table 1.** EPS and FN Errors

Experiment	Total FN	FN in EPS	FN not in EPS	% FN in EPS	n	p-value
Experiment 1 - fMRI	52	37	15	71%	9	P < 0.01
Experiment 1 - fNIRS	39	29	10	74%	8	P < 0.01
Experiment 2 - fMRI	29	21	8	72%	6	P < 0.01

### (3) Experiment 3: Observational Study of Radiologists in the Actual Clinical Work Setting

For the 9 radiologists who were observed using fNIRS in the actual clinical setting while engaged in their normal interpretive work activities, all participants were found to be in the EPS, on-average, 23.5% (range 17-33%, with SD = 5.38) of their working time, as summarized in Table 2. This EPS prevalence was consistent with the other two experiments in which the participants performed visual and attentional tasks.

**Table 2.** Fraction of Time in EPS

Experiment	Range	Mean	S.D.
Experiment 1	24-35%	30.4%	3.8%
Experiment 2	24-34%	28.9%	4.8%
Experiment 3	17-33%	23.5%	5.4%

These EPS prevalence rates are also in concordance with the existing literature, in which subjects had been found to be in the EPS 20-30% of the time during demanding mental tasks.^2^

**Figure 8.**
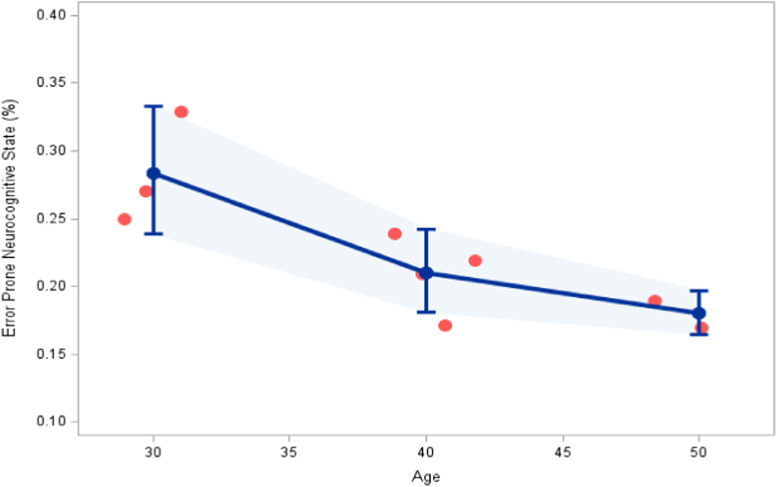
Prevalence of EPS by age, as determined from *f*NIRS of 9 subjects during their normal tasks, performed in the actual clinical setting, showing anti-correlation of EPS prevalence with age (p<0.001). Error bars = 95% CI

There was also a significant anti-correlation of the EPS fraction by subject age (p<0.001), as shown in Figure 8. This finding also aligns with past data,^26,27^ suggesting that older adults are off-task less frequently than younger adults. This observed trend is consistent with similar observations in other populations seen in prior studies,^28^ however its etiology and significance is uncertain; it may partially help to explain prior data showing that older radiologists are less susceptible to fatigue than are younger radiologists.^29^ The difference could be due to these subjects having longer experience, due to having had more years of practice, or it could be that the network fluctuations that lead to EPS actually represent a normal, physiological process of higher human mental functioning and the observation that EPS episodes occur less frequently in older persons may actually represent a feature of brain senescence. It does not appear to be a cohort bias.

No attempt was made during this experiment to determine whether any interpretive errors were made by any of the subjects during the period of observation. Future research is needed to examine the relationship between EPS events occurring in the actual clinical setting and their impacts on diagnostic accuracy.

Table 3 summarizes the key results of each of the three experiments.

**Table 3.** Results Summary

Experiment	Key result
Experiment 1	Continuous-attention task using fMRI and fNIRS simultaneously. Participants were found to be in the EPS between 24-35% of the time on-task; 71% of FN errors occurred during an episodes of EPS. The two brain imaging modalities, fMRI and fNIRS had a 73% concordance for detection of EPS.
Experiment 2	Visual search task using fNIRS and Eye-tracking. Subjects were found to be in the EPS 24-34% of the time on-task. FN errors were approximately threefold more likely during an episode of EPS. These detection errors were observed whether or not the participant foveated the target.
Experiment 3	Observational study using fNIRs in the participants’ actual clinical setting as they did their usual clinical work. Participants were found to be in EPS between 17-33% of the time. Prevalence of EPS was inversely correlated with age (with a high level of statistical significance).

## Discussion

While subject numbers are low in this pilot study, these results generally support the hypothesis that an error-prone state (EPS) arising from non-volitional episodic co-activation of the nominally-opposed DMN and FPN networks—which appears to occur on a stochastic basis—was associated with a majority of the observed perceptual errors in our radiologist subjects. FN (miss) errors were found to be significantly more likely in the presence of this neurocognitive EPS than at other times, with an approximately threefold increase in the risk of error at these times. The relationship between the observed EPS and false-negative errors was observed in both a search task and a continuous-attention challenge task. Our results also provide proof-of-concept that the EPS can be detected unobtrusively in the clinical setting.

We suggest that this involuntary neurocognitive human factor may be a factor in radiologists’ perceptual errors in practice, which also appear to occur randomly and without the awareness of any attentional lapse or departure from usual image evaluation on the part of the operator. The prevalence of EPS in our relatively small sample is also in keeping with the observed prevalence of perceptual errors in actual radiological practice, which has remained essentially unchanged since first measured in the late 1940s.^6^ This mechanism of error would also account for the observed higher error rates occurring when the case mix is artificially “enriched” with a larger fraction of positive cases than are usually seen in routine practice.

The EPS episodes we observed, using two independent measures of BOLD signal, were found to be variable in duration and frequency, but occurred with similar prevalence across all tasks studied, including when radiologists performed their usual image interpretation tasks in their actual clinical practice work settings. We found that the unobtrusive, portable technique of fNIRS correlated with the “gold standard” of fMRI to a high degree, with a 73% concordance in frequency, timing and duration of EPS events and concordance on 67% of FN errors observed during EPS, using the analytical criteria described. In this way, our study also has demonstrated the feasibility of using the unobtrusive method of fNIRS to reliably and reproducibly (if imperfectly) detect the EPS in the actual clinical work setting, providing a pathway for intervention to reduce the risk of perceptual errors in practice.

Our finding of a high level of concordance between fMRI and fNIRS is consistent with very recently published results using concurrent whole-brain fMRI and fNIRS using similar methods in a different experimental context.^30^ We would not have expected a perfect correlation between the two modalities, since fNIRS assesses only two (superficial) brain nuclei compared to several (including both superficial and deep cortical areas) for fMRI, however the high level of correlation that we observed between the two modalities should be sufficient to allow the development and testing of intervention strategies based on fNIRS in future work. Further, there is some evidence that certain subsystems within the DMN, including the medial PFC, may have a greater contribution to attentional lapses and thus the risk of perceptual error in radiology than others, which, if correct, would make the translation approach we envision potentially even more effective.^31,32^

Distinct interactions between various individual components of the FPN and DMN, especially the PFC components, have also recently been implicated in mediating people’s level of conscious awareness.^33^ It is worth noting that some prior published literature has used the term “mind-wandering” interchangeably with the brain activation state we have chosen to call EPS, and there is likely significant overlap. The distinction is mostly behavioral, as without an interruptive query/experience sampling, we cannot determine to what extent an observed EPS episode might correspond to mind-wandering. EPS does, however, appear to be distinct from the phenomenon of “inattentional blindness,” in that the latter appears to relate to visual salience of the target and contrast between the target and distractors.

We have focused on FN “miss” errors, as these account for the overwhelming majority of radiologists’ errors in practice,^2-4^ and indeed we have again found FP errors to be quite rare and, as expected, unrelated to the EPS neurocognitive mechanism. A perhaps surprising result was noted with the addition of eye-tracking to our visual search task: it would appear that there is no difference, from the standpoint of the EPS, between FN errors where the subject foveated the target and when they did not. In other words, we did not detect a difference between “not looking” and “looking without perceiving.”

## Limitations of Our Pilot Study

The key limitation of this preliminary work is the sample size, especially considering the small number of error events per subject, and limited participant demographics. There is currently an international workforce shortage of radiologists^34^ that makes recruitment of participants challenging. The available participants at our institution were predominately male and right-handed, which is a limitation for our analysis, as there may be gender and handedness differences in EPS. To increase confidence in the significance of our results, larger sample size will be needed. In future work, we plan to recruit participants from at least two institutions, with greater gender balance, ethnic and cultural diversity, and to include trainees, untrained laypersons, and more individuals who are left-handed.

Other limitations include our recruitment of subjects who are all highly experienced radiologists, as results from individuals with less training and experience (*e.g.,* residents or fellows) may differ. Finally, although we did not strictly control for time of day and thus have not addressed the potentially confounding effect of fatigue, which is known to affect radiologists to differing degrees across the lifecycle^29,35^ our subject runs were confined to the late morning and early-to-mid afternoon (see technical supplement) in an attempt to minimize this potential confounding variable. Future work is planned to more systematically evaluate the effect of fatigue on the prevalence of EPS and resultant FN error. It is likely that the prevalence of EPS would be increased overall under fatigue conditions; an intriguing question would be whether the age-gap would widen. This would be a fruitful avenue for future study.

Finally, it should be stated that the key criteria used for identification of the EPS on each of our functional brain imaging modalities (fMRI and fNIRS) required selection of threshold signal detection values, in the form of a Pearson correlation cutoff of 0.4 for fMRI and amplitude thresholds for fNIRS. Thresholds were chosen to optimize both sensitivity and specificity for EPS detection and correlation with FN errors, as discussed above. Raising the thresholds would increase specificity and lowering them would increase sensitivity, in a “trade-off” fashion, analogous to the reasoning that underpins ROC analysis. While the effects we observed were robust across a range of correlation thresholds, as noted above, the choice represents a compromise. But this analytical approach also carries a potential risk of dichotomizing a phenomenon which could instead represent a continuum of brain states rather than two discrete neurocognitive states. Further study with larger subject (and event) numbers will allow deeper exploration of the phenomenon we are describing to help to constrain the EPS hypothesis. Further, our pilot study, which depended on observational and correlative design in a small number of subjects, while strongly suggestive, is not able to definitively establish a causal role of EPS in producing perceptual errors. Greater certainty regarding the apparent association between EPS and look-but-fail-to-see errors will be greatly strengthened if alternative interpretations, *e.g.,* fatigue, cognitive-load limitations, and variations in task difficulty, can be more fully controlled in a much larger and diverse participant pool in future work.

### Clinical Applications/Intervention Pathways

Additional work will be needed in order to translate this work to the clinical setting. For example, one could envision a system for real-time monitoring of radiologists using a wearable fNIRS-based device providing input to a machine-learning algorithm that can analyze the output of the fNIRS system and detect the presence of the EPS in real-time, and provide some sort of feedback to the operator at the point-of-care. Engineering challenges would include development of a wearable detector device that is sufficiently comfortable and unobtrusive as to be readily tolerated by radiologists as they worked, including being able to connect wirelessly to a computer for rapid analysis of the output so that there was no restriction of movement. A machine-learning algorithm could be additionally trained to correlate the timing of detected EPS episodes to the clinical images that are being contemporaneously viewed, so those can be re-presented to the radiologist for a second-look in the absence of EPS. Such a system might be expected to be able to dramatically reduce perceptual errors in Radiology if the engineering challenges could be surmounted. To enhance detection accuracy and reduce false-detection events, a multi-modal approach could be applied, such as combining fNIRS data with webcam-based eye-tracking, which is known to be technically feasible in the clinical setting.^36-38^ The feasibility of such a multimodal approach has very recently been demonstrated combining neurophysiological monitoring using EEG combined with facial video features.^39^

By combining these diverse detection modalities, we may reasonably expect to increase both the sensitivity and specificity for detection of the high-risk neurocognitive state, in order to allow rapid biofeedback to be presented to the user. Such feedback may, in turn, allow radiologists to make behavioral changes in order to reduce the risk of perceptual error resulting from this mechanism. Further work will be needed to determine whether interventions based on detection of the EPS from real-time monitoring, aided by deep-learning algorithms, will be effective to reduce the risk of radiologists’ perceptual errors in practice. Additionally, radiologists’ training and continuing education could be tailored to help them to understand and utilize this type of feedback as a means of mitigating error risk.

## Conclusions

In conclusion, further research is also needed to better establish the relationship between perceptual error and brain network states in a larger sample of radiologists throughout all phases of the life and career cycle, and with a broader range of tasks with varying levels of task difficulty. It would also be useful to compare trained, expert radiologists to laypersons, in order to determine the effect of the radiologists’ extensive training and experience on the phenomena observed. It is encouraging that our results are both highly significant and align well with previously published findings in laypersons performing a variety of visually-based tasks, suggesting that we are observing a normal feature of human higher mental function. The anti-correlation of EPS prevalence with age that we observed may relate to the radiologists’ intensive training and long experience in performing concentrated visual search and discrimination tasks, *i.e.,* a “practice effect,” producing a lesser degree of mental deviation from the task with increasing years of experience vs. a feature of brain senescence, as discussed above. Interdisciplinary collaboration involving radiologists, visual perception scientists, evolutionary and cognitive psychologists and neuroscientists with expertise in functional brain imaging would be ideally suited to addressing these questions.

The results of this pilot study, despite small subject and event numbers, suggest that a readily detectable biomarker of error risk in radiologists may exist, a neurocognitive error-prone state, which can be reliably detected using unobtrusive technology and could conceivably be used as a biomarker in real-time, to provide for point-of-care monitoring, operator feedback and, potentially, error-risk reduction. If ultimately successful in clinical translation, this approach could be the first to meaningfully address the phenomenon of perceptual errors in radiologists, which were first described in 1949 and which have been intractable to all prior attempts at mitigation.

## Appendix

### Abbreviations

CAT task: a visual task requiring continuous operator attention
DMN: Default Mode Network
FPN: Frontoparietal Network
EPS: The “Error-Prone State” observed with simultaneous co-activation of the DMN and FPN, which are generally opposed
*f*MRI: functional MRI
fNIRS: functional near-infrared spectroscopy, an unobtrusive functional brain imaging modality
FN: false-negative errors
FP: false-positive errors
Foveated, foveation: When a participant’s eye movements cause a visual target to lie within the central, foveal vision. This confirms that the participants looked directly at the target
PFC: Prefrontal Cortex
Visual Search task: a visual task requiring the participant to search a visual field for a solitary target placed in random locations along with numerous similar-appearing distractors.
